# *Glomus* sp. and *Bacillus* sp. strains mitigate the adverse effects of drought on maize (*Zea mays* L.)

**DOI:** 10.3389/fpls.2022.958004

**Published:** 2022-08-17

**Authors:** Emilia Wilmowicz, Agata Kućko, Kalisa Bogati, Magdalena Wolska, Michał Świdziński, Aleksandra Burkowska-But, Maciej Walczak

**Affiliations:** ^1^Chair of Plant Physiology and Biotechnology, Faculty of Biological and Veterinary Sciences, Nicolaus Copernicus University, Toruń, Poland; ^2^Department of Plant Physiology, Institute of Biology, Warsaw University of Life Sciences-SGGW, Warsaw, Poland; ^3^Department of Environmental Microbiology and Biotechnology, Faculty of Biological and Veterinary Sciences, Nicolaus Copernicus University, Toruń, Poland; ^4^Department of Cellular and Molecular Biology, Nicolaus Copernicus University, Toruń, Poland; ^5^Bacto-Tech Sp. z o.o., Toruń, Poland

**Keywords:** *Bacillus*, drought tolerance, *Glomus*, maize, pectin, reactive oxygen species, cell wall

## Abstract

Maize (*Zea may*s L.) is an economically important source of food and feed. This species is highly sensitive to drought, which is the most limiting factor for the biomass yield of a crop. Thus, maize cultivation methods should be improved, especially by environment-friendly agricultural practices, such as microorganisms. Here, we provide evidence that *Glomus* sp. and *Bacillus* sp. modulate maize response to drought. Inoculation of maize seeds by these microorganisms restored the proper photosynthetic activity of the plant under drought and stabilized the osmoprotectant content of the leaf. The beneficial effect of *Glomus* sp. and *Bacillus* sp. was also related to the stabilization of cell redox status reflected by hydrogen peroxide content, antioxidant enzymes, and malondialdehyde level in leaves. As we revealed by several methods, shaping maize response to drought is mediated by both microorganism-mediated modifications of cell wall composition and structure of leaves, such as downregulating pectin, affecting their methylation degree, and increasing hemicellulose content. Overall, we provide new information about the mechanisms by which *Glomus* sp. and *Bacillus* sp. induce drought tolerance in maize, which is a promising approach for mitigating abiotic stresses.

## Introduction

Drought is one of the greatest threats to modern agriculture. In different parts of the globe, changes evoked by anthropogenic environmental pressure may result in a significant reduction in the yield of many crops, such as *Zea mays* L. (maize). This could consequently lead to a global humanitarian crisis, given that maize is one of the most widely cultivated grain crops worldwide. Compared to rice and wheat, the nutrient composition of maize consists of approximately 10% protein, 72% starch, 4% fat, several B vitamins, essential minerals along with fiber, and other energy-dense components of food (Dale and Fuller, 1982; Nuss and Tanumihardjo, 2010). The United States, Brazil, and China are the top maize-producing countries in the world. This species provides key substrates used in many industries, including food, paper, and fodder, as well as has great potential in bioenergy production (Tanumihardjo et al., 2019). Furthermore, gluten-free cornmeal is widely used in the allergic diet, while starch corn seeds are a valuable component for feed. The oil produced from the germ of corn kernels is a source of beneficial unsaturated fatty acids. Importantly, corn seeds contain more polyphenols and show stronger antioxidant properties compared to other grains, e.g., rice, wheat, or oats (Dewanto et al., 2002; Siyuan et al., 2018). A major portion of maize production is utilized in ethanol fuel. This is commonly used as a motor fuel, as a biofuel additive replacement for gasoline.

Maize yield, particularly at critical growth periods, can be affected by water stress conditions. Prolonged drought stress resulted in the reduction of leaf size, reduced the vitality of seedlings, and increased death of embryos after pollination, thereby leading to a drastic decline in crop yield (Kakumanu et al., 2012; Mao et al., 2015; Chen et al., 2016). To fulfill the requirements for food and nutrition all over the world, the production of maize cereal has to be improved and protected under drought conditions. For this reason, several approaches and biofertilizers are investigated to enhance drought tolerance and promote plant growth (Ullah et al., 2017, 2019). The present methods of agricultural crop production, for instance, the use of improper chemical fertilizers and pesticides, may lead to harmful production of greenhouse gases and consequently environmental and human health problems (Shams et al., 2017).

Therefore, microbes that produce beneficial compounds can protect the plants and provide a vital solution for a sustainable and environment-friendly agricultural practice for the improvement of crop yield under unfavorable environmental conditions, such as drought (Glick, 2014; Ullah et al., 2019). This can be achieved by the synthesis of natural formulations of microbiological origin. Most of the current formulations are composed of single strains of microorganisms or their consortia. Among them, special attention should be focused on *Bacillus* sp. (bacteria) and *Glomus* sp. (fungus). To date, several physiological aspects of both microorganisms have been analyzed in plants like *Zea mays* (Vardharajula et al., 2011), *Capsicum annum* (Lim and Kim, 2013), *Triticum aestivum* (Kasim et al., 2013), *Lactuca sativa* (Ruiz-Lozano et al., 1995b), and *Cucurbita pepo* (Harris-Valle et al., 2018). Generally, *Bacillus* sp. is a soil-living bacteria. Its spores are present in the environment until the optimal conditions for proliferation occur (Earl et al., 2008). *Bacillus* sp. can exist in the rhizosphere as plant growth-promoting rhizobacteria (PGPR) or as a symbiotic bacteria. It was demonstrated that they are able to produce phytohormones, such as gibberellins, cytokinins, auxins, and polyamines, and in this way directly affect the growth of root and root hairs (Joo et al., 2004; Xie et al., 2014). Most studies regarding *Bacillus* sp. focus on their role as PGPR; however, their involvement in the mediation of plant drought responses should be particularly investigated, given the high sensitivity of crops to this stress factor. It has been shown that *Bacillus subtilis* can increase water status, photosynthetic activity, and nutrient availability; promote the accumulation of osmoprotectants including sugars and amino acids; and accelerate the production of antioxidants (Arkhipova et al., 2007; Ashraf and Foolad, 2007; Vardharajula et al., 2011). There are also reports indicating that *Bacillus* sp. mediates drought resistance in *Brachypodium distachyon* through the stimulation of the expression of drought-responsive genes, accumulation of sugars and starch in leaves, and affecting DNA methylation (Gagné-Bourque et al., 2013, 2015). *Bacillus* sp. improves the growth of *Phleum pratense* L. under drought by increasing the shoot and root biomass, photosynthetic rate, and stomatal conductance, and accumulation of saccharose, fructans, and amino acids (asparagine, glutamic acid, glutamine, and non-protein amino acid γ-aminobutyric acid) (Gagné-Bourque et al., 2016).

The mediation of plant stress responses by *Glomus* sp. has also been documented. These fungi positively influenced plant growth, mineral uptake, CO_2_ exchange rate, water efficiency, transpiration, stomatal conductance, photosynthetic efficiency, and proline accumulation (Ruiz-Lozano et al., 1995a). Additionally, increased content of soluble proteins and higher activity of antioxidant enzymes have been noted in plants under the action of *Glomus* sp. (Wu et al., 2007; Sohrabi et al., 2012; Gong et al., 2015). A common mechanism of a plant’s response to environmental cues is the disrupted balance between the production and scavenging of reactive oxygen species (ROS). One of the most toxic ROS that is formed in every cellular compartment is superoxide anion radical (O_2_^•⁣–^), which is quickly scavenged by superoxide dismutase (SOD) into H_2_O_2_ (Mittler, 2002; Hasanuzzaman et al., 2020). This, in turn, is inactivated by the action of catalase (CAT). ROS play a key role in the modification of the cell wall, which is the first site of perception of abiotic stress and simultaneously constitutes the first protective barrier against its effects. The primary cell wall mainly consists of hemicelluloses, celluloses, and pectins (Harholt et al., 2010; Atmodjo et al., 2013). It has been shown that drought can affect its structure by altering the proportion of different components, leading consequently to decreased extensibility and plant vitality under conditions of osmotic stress (Le Gall et al., 2015). Therefore, we aimed to determine whether inoculation of maize seeds with *Glomus* sp. or *Bacillus* sp. can modify the leaf cell wall structure under drought conditions and thereby mitigate the negative impact of water deficit on the above-ground part of the plant, which guarantees reproductive success and high yield.

In the beginning, we verified whether experimental conditions are sufficient to induce stress-related physiological responses. For this purpose, we measured photosynthetic activity, analyzed leaf structure, level of osmoprotectant (a marker of oxidative stress), the level of selected ROS, and activity of antioxidant enzymes under drought conditions and post-inoculation with *Glomus* sp. or *Bacillus* sp. Modifications in the cell wall structure were evaluated by determining the pectin methylation and hemicellulose content. Collectively, our results show that both the tested microorganisms affect the cell wall composition and reduce the adverse effects of drought in the leaves of *Z. mays*.

## Materials and methods

### Inoculum preparation and growth of bacteria

*Glomus* spp. and *Bacillus* sp. were provided by Bacto-Tech sp. z o.o. (Poland). *Glomus* sp. (the mycorrhizal inoculum) was composed of peat, spores, hyphae, and root fragments of *Plantago major* L. This mixture was stabilized by freeze-drying. Furthermore, *Bacillus* sp. were grown in nutrition broth in a 3 L bioreactor for 7 days (26°C) and then centrifuged for 5 min at 10,000 x*g*. The obtained biomass of bacteria was stabilized by freeze-drying. The number of bacteria in the obtained sample was 10^11^ colony-forming units/g (CFUs/g).

### Seeds treatment, stress application, and plant cultivation

The plant material used in this study was maize (*Zea mays* L.). Seeds were moistened with water. Then, they were divided into three groups: the first group did not receive any inoculation, the second group was inoculated with the powder composed of fungal strains (*Glomus* sp.), and the third group was inoculated with the powder composed of bacterial strain (*Bacillus* sp.). We used 800 mg of inoculation powder per 10 seeds. After that, seeds were sown in pots filled with soil, and the plants were cultivated in the phytotron chambers under controlled light and temperature conditions (22 ± 1°C, 110 μmol m^–2^s^–1^, and cool white fluorescent tubes). Plants were watered for 2 weeks in optimal 70% soil water holding capacity (WHC). Subsequently, maize seedlings inoculated with bacteria were cultivated for 4 weeks (∼31 days) in 25% WHC. Non-inoculated plants were divided into two groups: the first one was cultivated for 2 weeks in 25% WHC, while the second one was grown in well-watered conditions (control). WHC calculations were made according to Chauhan and Johnson (2010) with minor modifications described by Wilmowicz et al. (2019). After 6 weeks, plants were subjected to biometric analysis and photosynthesis-related parameters. Fresh tissues were used for *in vivo* histochemical staining. Additionally, leaves were collected for further biochemical experiments, frozen in liquid nitrogen, and stored at −80°C. In turn, for all microscopy assays, leaf sections were immediately fixed.

### Chlorophyll fluorescence parameter (Fv/Fm)

The maximum quantum efficiency of PS II (Fv/Fm) was measured using a portable modulated OS-30P (Opti-Sciences, Inc., Hudson, NH, United States) according to the method described by Weng (2006). For each measurement, all the leaves from the plant were clamped at the center of the leaf clip holder for dark adaptation (30 min). Analysis was made using five plants. The results are presented as mean ± SE (*n* = 3).

### Proline determination

Proline was analyzed according to the method of Ábrahám et al. (2010). In brief, leaves (∼0.5 g) were grounded in the presence of 3% sulfuric acid (5 μl/mg fresh weight), and then the extract was centrifuged (15,000×*g*, 5 min). The reaction mixture containing 100 μl of supernatant, 0.1 ml of 3% sulfosalicylic acid, 0.2 ml of glacial acetic acid, and 0.2 ml of acidic ninhydrin was prepared and incubated at 96°C for 30 min. Then, 1 ml of toluene was added to the samples, and the absorbance of the extract was read at 520 nm. The content of proline was calculated in reference to a prepared calibration curve. Values are expressed as μg proline g^–1^ fresh weight.

### Reactive oxygen species and reactive oxygen species-related enzyme analysis

Apoplastic release of O_2_^•⁣–^ was visually detected by incubating hand-cut leaf sections according to Rodríguez et al. (2004). We used nitroblue tetrazolium (NBT) as it reacts with O_2_^•⁣–^. A blue formazan precipitate was formed after the reaction. Briefly, tissue fragments were transferred to the tubes containing 0.01% NBT in 10 mM PBS (pH 7.8). After incubation in the dark (2 h at 30°C), tissues were kept in 10 mM PBS (pH 7.8) and photographed.

H_2_O_2_ was analyzed following the method of Loreto and Velikova (2001), which involves the oxidation of KI by H_2_O_2_ in an acidic solution. In brief, the collected leaves (∼0.5 g) were grounded with 3 mL of 1% (w/v) trichloroacetic acid (TCA). The obtained homogenate was centrifuged for 15 min (14,000×*g*, 4°C). After that, 0.75 ml of the supernatant was mixed with 0.75 ml of 10 mM K-phosphate buffer (pH 7.0) and 1.5 ml of 1M KI. The absorbance of the mixture was recorded at 390 nm. The concentration of H_2_O_2_ was calculated by comparing it with a standard curve and expressed as μmol mg^–1^ fresh weight.

Protein extracts for enzymatic activities were prepared as follows. Frozen leaves (∼0.5 g) were grounded with 5 ml of extraction buffer (50 mM K-phosphate buffer (pH 7.6) and 0.1 mM Na-EDTA). The homogenate was centrifuged (12,000×*g* for 15 min), and the obtained supernatant was used for SOD and CAT analyses. The total SOD activity was assayed according to Giannopolitis and Ries (1977).

The total CAT activity was determined by monitoring the decrease in absorbance at 240 nm (following the decomposition of H_2_O_2_) following the method of Cakmak and Marschner (1992). The reaction was started by adding 50 μL of the substrate to a mixture composed of 50 mM K-phosphate buffer (pH 7.0) and 10 mM H_2_O_2_. The results are expressed as μmol of H_2_O_2_ s^–1^ g^–1^ fresh weight.

### Staining and quantification of pectin and hemicellulose determination

Fresh leaf fragments were incubated for 30 min with 0.02% (w/v) ruthenium red ([(NH_3_)_5_Ru-O-Ru(NH_3_)_4_-O-Ru(NH_3_)_5_]Cl_6_) to determine unesterified pectin (Sabba and Lulai, 2002). Additionally, fragments of leaves (∼0.1 g) were immersed in 0.5 mmol L^–1^ of CaCl_2_ solution and washed two times with water. Pectins were isolated and quantified according to Liu et al. (2019) with slight modifications, as described in our previous work (Florkiewicz et al., 2020). The hemicellulose content was analyzed following the methodology described by Florkiewicz et al. (2020).

### MDA quantification

Frozen leaves (∼0.5 g) were grounded in a chilled mortar, and further steps of malondialdehyde (MDA) determination were followed as described by Hodges et al. (1999) with some modifications, as presented in our recent paper (Kućko et al., 2022).

### Microscopy sample preparation and histological assay

The tissue fragments were excised from the central area of the leaves and were immediately fixed in 4% paraformaldehyde and 0.2% glutaraldehyde prepared in 1 × phosphate-buffered saline (PBS, pH 7.2). After 12 h of incubation at 4°C, tissues were dehydrated, supersaturated, and embedded in BMM resin (methyl methacrylate, butyl methacrylate, 10 mM dithiothreitol, and 0.5% (w/v) benzoin ethyl ether; Fluka, Buchs, Switzerland) as described previously (Wilmowicz et al., 2016). Then, an Ultracut microtome (Reichert-Jung, Germany) was used for the preparation of semi-thin sections (1 μm). The sections were subjected to general histological observations. First, toluidine blue dye was applied at a concentration of 0.05% for 10 min. The second experiment involved staining for 30 min with 0.02% (w/v) ruthenium red (Sabba and Lulai (2002). The obtained samples were analyzed using a microscope (LM Zeiss Axioplan, Oberkochen, Germany) equipped with a ProGres C3 digital camera.

### Immunofluorescent experiments for detection of high- and low-methylated pectins

Semi-thin sections, obtained as described in the previous section, were subjected to immunodetection. We analyzed low- and non-methylated pectins (31–40%), and high-methylated pectins (15–80%) using JIM5 and JIM7 antibodies, respectively (Willats et al., 2000). Our protocol published previously (Florkiewicz et al., 2020) was adopted. Additionally, for the visualization of nuclei, the sections were incubated with DAPI following the method of Florkiewicz et al. (2020). The samples were finally observed under a fluorescent microscope (DM6000B, Leica, Wetzlar, Germany).

### Statistical analysis

Statistical analysis and presentation of the obtained results were performed using MS Excel 365 (Microsoft) and SigmaPlot 2001 v. 5.0. Each result was presented as the mean ± standard error (SE) of at least three replicated measurements (*n* = 3). The significant differences between the tested variants were compared by Student’s *t*-test.

## Results

### Verification of stressful conditions in *Zea mays* leaves

**FIGURE 1:**
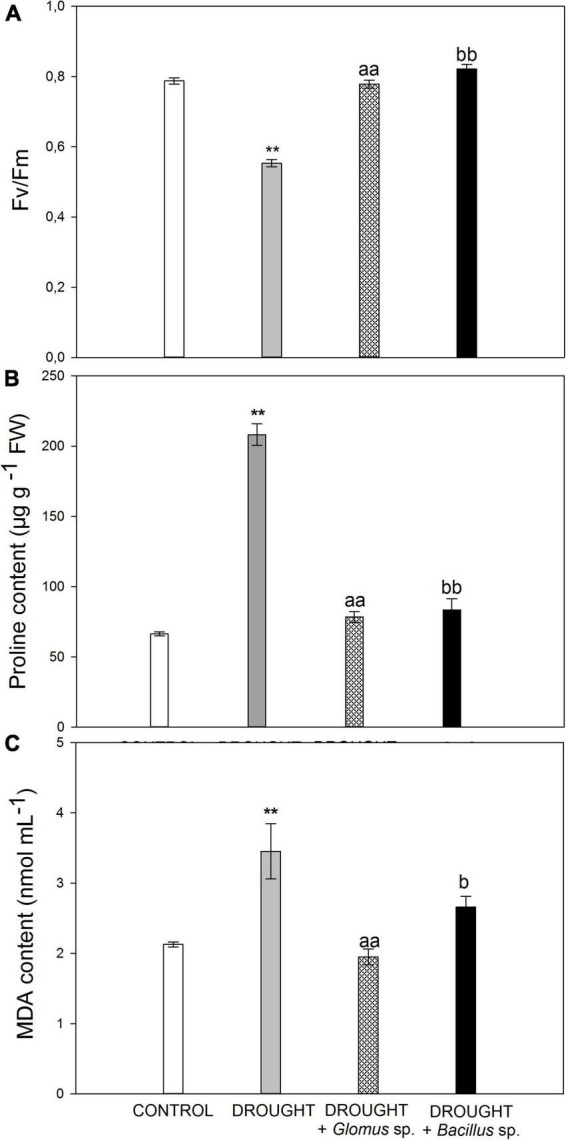
Values of maximum quantum yield efficiency of PSII (Fv/Fm) **(A)**, proline **(B)**, and MDA **(C)** content in the leaves of *Zea mays* subjected to drought conditions or inoculated with the *Glomus* sp. or *Bacillus* sp. prior to planting and drought treatment. Values for Fv/Fm are presented as mean ± SE (*n* = 5), while values for proline and MDA concentration are presented as mean ± SE (*n* = 3). Significant differences for drought-treated plants versus control are **P < 0.01; for drought + *Glomus* sp. vs. drought *^aa^P* < 0.01; for drought + *Bacillus* sp. vs. drought *^b^P* < 0.05, *^bb^P* < 0.01.

In the beginning, we aimed to verify whether the drought stress conditions were sufficient to induce the response in *Z. mays* plants. The reduced water potential limits the transpiration and CO_2_ flow to the cell, caused by the closing of the stomata, and consequently leads to declined photosynthetic rate (Lawlor and Tezara, 2009). The maximum quantum efficiency of PSII is reflected by the chlorophyll fluorescence parameter Fv/Fm, which is widely used to analyze the stress response in plants (Murchie and Lawson, 2013). Here, we show that this parameter decreased in the leaves of *Z. mays* subjected to drought conditions (Figure 1A), reaching a value of ∼0.55. In contrast, Fv/Fm values in the stressed plants developed from the seeds treated with *Bacillus* sp. or *Glomus* sp. were similar to those observed in the untreated control (Figure 1A).

One of the strategies involved in the defense mechanism of plants that is activated in drought conditions is the increased synthesis and accumulation of osmoprotectants, such as proline. These molecules are responsible for osmotic adjustment in cells, since they cause a decrease in water potential, which further improves plant tolerance to adverse environmental conditions (Ashraf and Foolad, 2007). To provide additional evidence for the drought action observed in *Z. mays* plants, we aimed to check the level of proline (Figure 1B). Control leaves accumulated ∼70 μg g^–1^ fresh weight, while the level of this osmoprotectant in the leaves collected from drought-stressed plants was almost higher by three times. When we subjected the plants to combined drought treatment, due to the inoculation of additional seeds prior to sowing, we observed a decrease in the content of proline when compared to the single drought treatment. Furthermore, the proline content in the samples subjected to the combined treatment was higher than that observed in the control. Water deficit in the soil leads to the generation of secondary stress-related compounds like ROS, which may affect the structure of nucleic acids, proteins, and lipids. Spontaneous action of ROS, as crucial molecules produced under stress conditions, can evoke lipid peroxidation and accumulation of toxic compounds (Gill and Tuteja, 2010). Among them, the most mutagenic is MDA, which has been recognized as a biological marker of oxidative stress (Shulaev and Oliver, 2006). The leaves of *Z. mays* cultivated under drought were characterized by an increased level of MDA (Figure 1C). However, when maize seeds were pretreated with *Bacillus* sp. or *Glomus* sp. strains and then the plants were subjected to drought, downregulation of MDA was observed.

### Effects of *Glomus* sp. or *Bacillus* sp. treatment on the redox state in *Zea mays* leaves under drought stress

**FIGURE 2:**
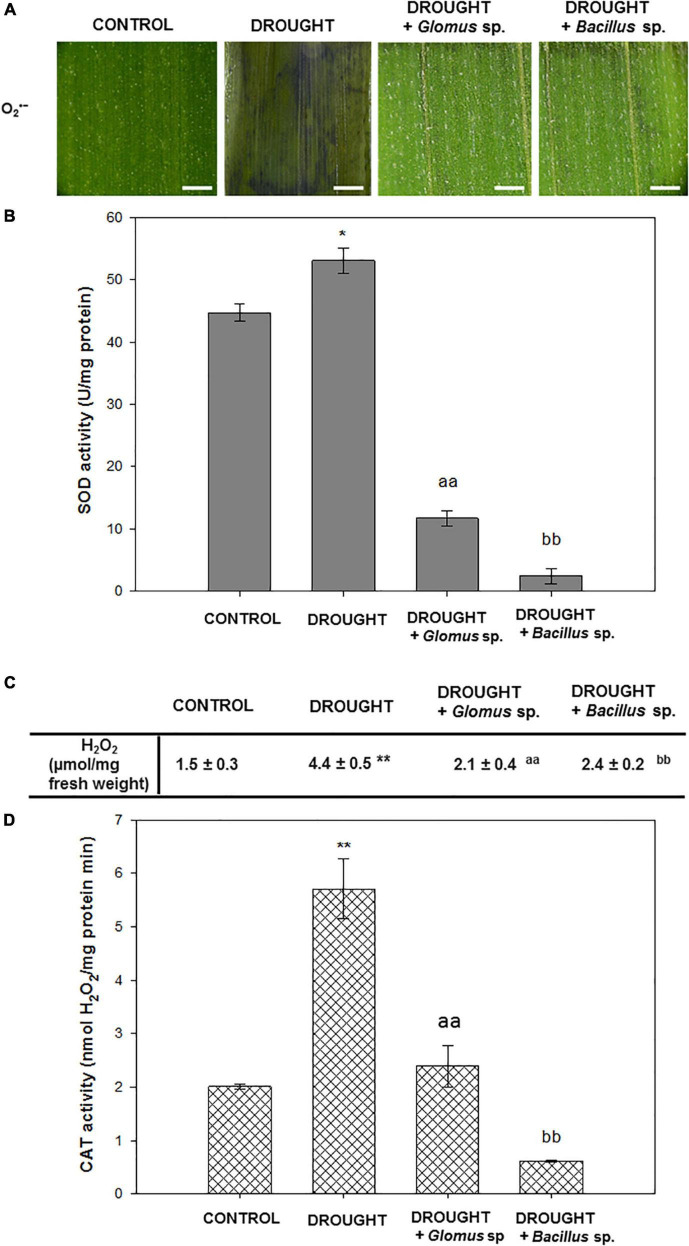
Seed inoculation combined with further drought stress affects ROS-related events in *Zea mays* leaves. Analyses have been done on leaves obtained from non-treated plants (control), drought-stressed plants, and drought-stressed *Z. mays* developed from seeds inoculated with *Glomus* sp. or *Bacillus* sp. Visualization of O_2_^•⁣–^ detected by histochemical staining **(A)**. The blue color corresponds to the presence of O_2_^•⁣–^. Bar = 1 mm. The activity of superoxide dismutase (SOD) **(B)**. The level of H_2_O_2_ **(C)** and activity of catalase (CAT) **(D)**. Significant differences for drought-treated plants vs. control are *P < 0.05, **P < 0.01; for drought + *Glomus* sp. vs. drought *^aa^P* < 0.01; for drought + *Bacillus* sp. vs. drought *^bb^P* < 0.01.

To check redox homeostasis in *Z. mays* leaves under the examined treatments, we performed staining for the O_2_^•⁣–^, which is one of the most toxic ROS, analyzed the activity of SOD responsible for O_2_^•⁣–^ dismutation, and then determined the level of H_2_O_2_ and the activity of the enzyme catalyzing its decomposition into H_2_O – CAT. NBT staining revealed that apoplastic O_2_^•⁣–^ was extensively accumulated in the leaves under drought conditions (Figure 2A). However, when stressed plants were pretreated with *Bacillus* sp. or *Glomus* sp., the observed drought-evoked effect was reversed. In these variants, O_2_^•⁣–^ was detected mainly along the leaf vascular system. Importantly, almost no staining was visible in the control tissue. The SOD activity was slightly modified by water deficit, and in this case, reached a value of ∼53 units/mg of protein (Figure 2B). When stressed plants were pretreated with *Bacillus* sp. or *Glomus* sp., the activity of SOD was significantly lower in comparison to non-treated and drought-stressed maize plants. A minimum value of ∼2 u/mg protein was noted for plants subjected to the influence of drought and *Bacillus* sp. Water deficit in soil caused a strong accumulation of H_2_O_2_ in *Z. mays* leaves (Figure 2C). In contrast, the leaves of plants exposed to drought and *Glomus* sp. were characterized by decreased content of this ROS. In this variant, the level of H_2_O_2_ decreased and maintained a value similar to that observed in the non-treated plants. A different relationship was observed in the drought-stressed plants in the presence of *Bacillus* sp., where the content of H_2_O_2_ was higher than that observed in the well-watered plants, however, does not reach the value noted in drought-stressed plants. A high amount of this compound in the leaves of drought-treated plants was correlated with the increased activity of CAT (Figure 2D). Treatment of the stressed plants with the *Glomus* sp. strain decreased the CAT activity to the control value; however, the activity was decreased by more than half when compared to that observed during the drought. The lowest CAT activity was found in the leaves of stressed plants pretreated with *Bacillus* sp.

### Effect of seed inoculation of *Glomus* sp. or *Bacillus* sp. on drought-evoked changes in the cellular structure of *Zea mays* leaves

**FIGURE 3:**
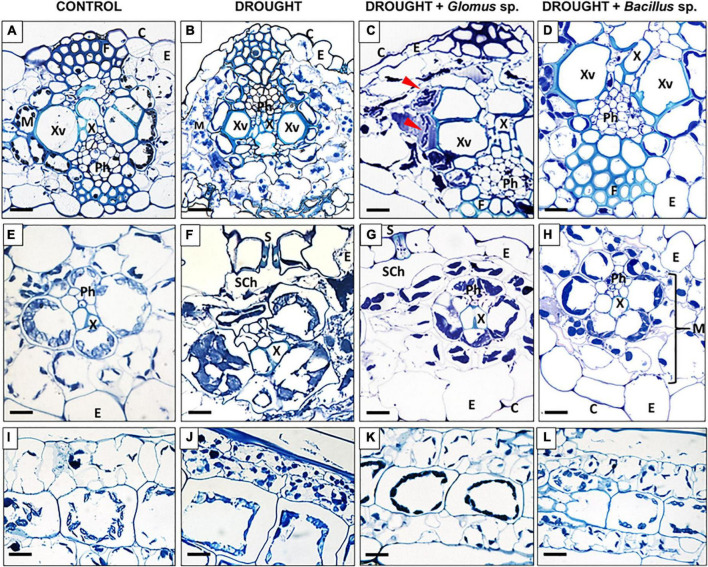
Light micrographs of cells of *Zea mays* leaves collected from plants subjected to the influence of *Glomus* sp. or *Bacillus* sp. and drought. Cross-sections were made from control **(A,E,I)**, drought-treated leaves **(B,F,J)**, and leaves collected from drought-treated plants, developed from seeds inoculated with *Glomus* sp. **(C,G,K)** or *Bacillus* sp. **(D,H,L)**. Tissue organization is described in images. The top panel shows a cross-section through the center area of the leaf, the middle panel shows the region of vascular bundles, and the bottom panel presents a longitudinal section of mesophyll cells. Possible microorganism presence was marked by red arrowheads **(C)**. C, cuticle; E, epidermis; F, fibers; M, mesophyll; Ph, phloem; S, stomata; Sch, substomatal chamber; X, xylem; Xv, xylem vessel. Bars = 15 μm.

Based on the fluctuations in the above-presented parameters. we can conclude that the application of these experimental conditions is sufficient to induce a response in the leaves. Moreover, specific cellular modifications in the leaf tissues were observed under the examined treatments (Figure 3). Water deficit in soil caused significant structural changes in leaves, such as degradation of mesophyll (Figure 3B), formation of smaller xylem vessels (Figure 3F), plasmolysis reflected by shrinkage of cells (Figures 3B,F), cell deformation (Figures 3B,F,J), and formation of several cytosolic aggregates (Figures 3B,F,J). Changes in the chloroplast location were particularly characteristic for drought-treated cells (Figures 3F,J). The structure of cells and tissues of leaves obtained from maize pretreated with *Bacillus* sp. before inducing drought conditions was similar to that observed in control (Figures 3D,H,L). More specifically, we observed well-developed vascular elements (Figure 3D), lack of plasmolysis symptoms (Figures 3D,H), properly developed mesophyll (Figure 3H), and chloroplasts positioned similar to the cells of untreated plants (Figure 3L), unlike that in drought-stressed plants (Figure 3J). Reversion of drought action by treatment with *Glomus* sp. was also manifested by cellular changes (Figures 3C,G,K). In these plants, the vascular elements were properly developed (Figures 3C,G). Nevertheless, microscopy analyses revealed that in several places protoplast gets detached from the cell wall (Figure 3C). Furthermore, some disorganization in the localization of chloroplast compared to the control section, particularly in the area surrounding the xylem, has been noted (Figure 3G). Interestingly, these areas indicated the possible presence of microorganisms (Figure 3C).

### The impact of seed inoculation of *Glomus* sp. or *Bacillus* sp. on the drought-triggered cell wall remodeling of *Zea mays* leaves

**FIGURE 4:**
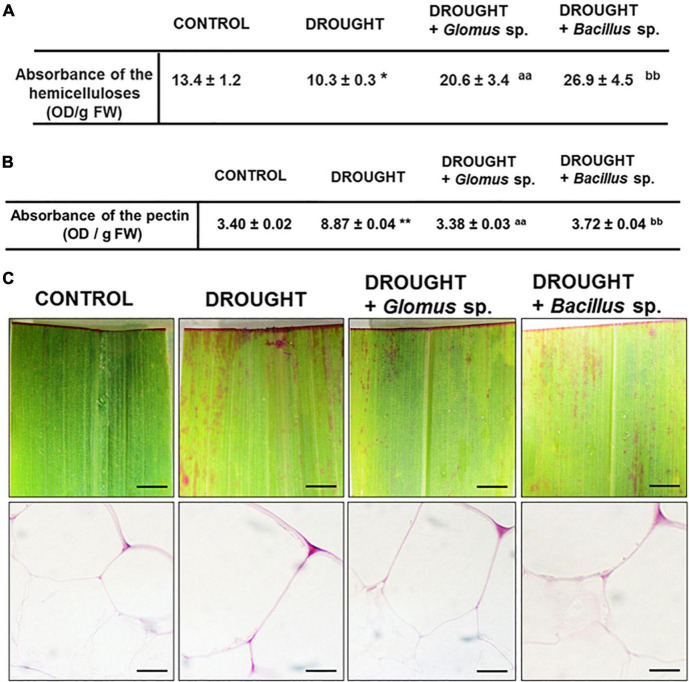
Effect of inoculation of *Glomus* sp. or *Bacillus* sp. on the pectin-related changes and hemicellulose content in maize growing under drought-stressed conditions. The analyses have been done on leaves from non-treated plants (control), drought-stressed plants, and drought-stressed maize developed from seeds inoculated with *Glomus* sp. or *Bacillus* sp. Quantitative analyses of the total pectin pool **(A)**. Visualization of de-esterified pectin (pink color corresponds to unesterified pectin presence) **(B)**. Bar = 1 mm (freshly stained leaves), 15 μm (cross-sections). Quantification of hemicelluloses **(C)**. Significant differences for drought-treated plants vs. control are *P < 0.05, **P < 0.01; for drought + *Glomus* sp. vs. drought *^aa^P* < 0.01; for drought + *Bacillus* sp. vs. drought *^bb^P* < 0.01.

Our histological analysis suggested that the cell wall might be modified under the action of drought stress. To examine the effect of both *Bacillus* bacterium and *Glomus* fungus strains on the events related to cell wall remodeling, we checked the content of hemicellulose in the maize leaves, which is the main carbohydrate in the middle lamella. As Figure 4A shows, under drought, the hemicellulose level reached a minimum value when compared to treatment groups. There was a significant increase in the content of this cell wall compound in plants inoculated with *Glomus* sp. or *Bacillus* sp. simultaneously cultivated under water deficit conditions. In such conditions, the level of hemicellulose was higher than observed in both control and plants exposed to drought.

In addition, we aimed to verify the possible changes related to the methylation level of cell wall pectin components. When maize was subjected to water deficit conditions, the total concentration of pectins drastically increased by almost three times when compared to well-watered plants (Figure 4B). Such an effect was not been observed in plants treated with drought and bacterial strains; the level of pectins was similar to that observed in the non-treated control plants. Further analyses have pointed out that the methyl esterification degree of pectins changes when plants are subjected to drought or exposed to bacteria. The intensity of red stain, which corresponds to the presence of unesterified pectin, was the highest in the drought-stressed leaves regardless of whether the plants were pre-inoculated with bacteria or not (Figure 4C).

**FIGURE 5:**
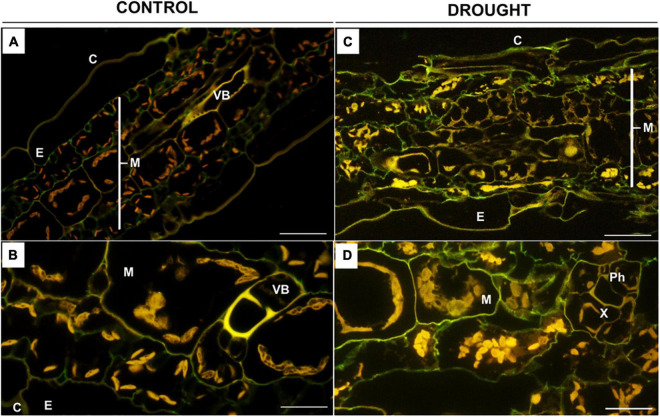
*In situ* immunolocalization of high-methylated homogalacturonans (HG) in *Z. mays* leaves under drought. Cross-sections of leaves from well-watered **(A,B)** and drought-stressed **(C,D)** plants were immunolabeled with the JIM7 antibody. Images were obtained by merged signals from JIM7, DAPI staining (blue color), and chlorophyll autofluorescence. Green fluorescence indicates the presence of high-methylated HG. C, cuticle; E, epidermis; M, mesophyll; Ph, phloem; Vb, vascular bundles; X, xylem. Bars = 50 μm **(A,B)**, 15 μm **(C,D)**.

**FIGURE 6:**
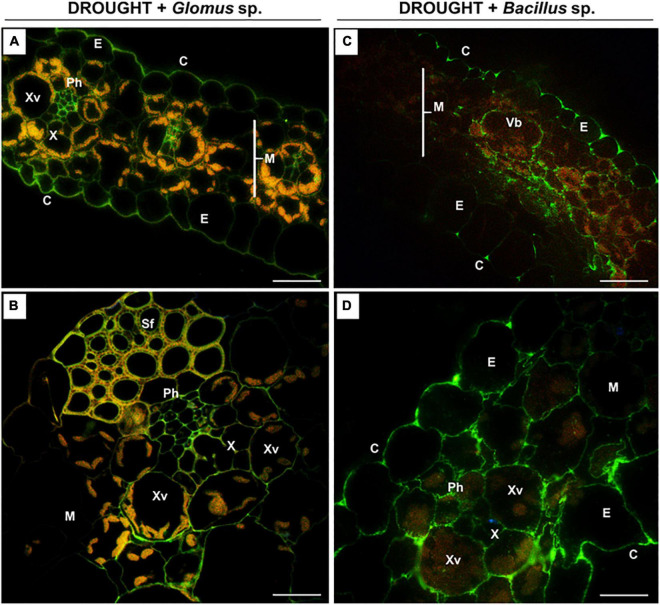
The influence of seeds inoculated with *Glomus* sp. or *Bacillus* sp. on the distribution of high-methylated pectins in the leaves of drought-stressed maize. Cross-sections of leaves from drought-stressed maize treated at the seed stage with *Glomus* sp. **(A,B)** or *Bacillus* sp. **(C,D)** were immunostained with the JIM7 antibody. Presented images were obtained by overlapping signals from JIM7, DAPI staining (blue color), and chlorophyll autofluorescence. Green fluorescence indicates the presence of high-methylated HG. C, cuticle; E, epidermis; M, mesophyll; Ph, phloem; Sf, sclerenchyma fibers; Vb, vascular bundles; X, xylem; Xv, xylem vessel. Bars = 50 μm **(A,B)**, 15 μm **(C,D)**.

**FIGURE 7:**
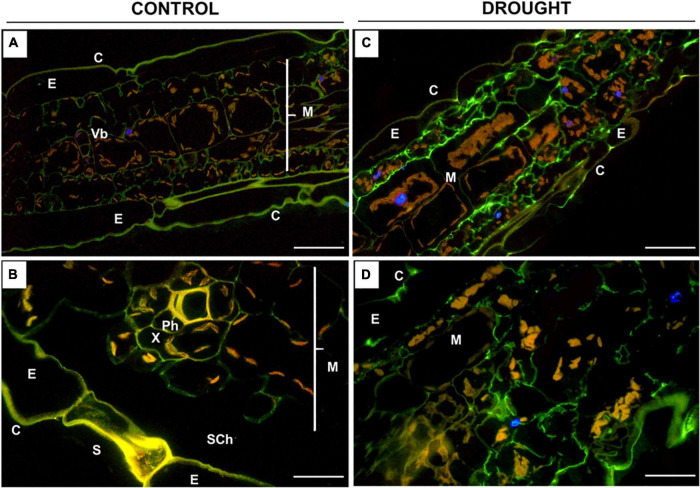
Immunolocalization of low-methylated homogalacturonans (HG) in *Z. mays* leaves under drought. Leaves from well-watered **(A,B)** and drought-stressed **(C,D)** plants were used for the preparation of cross-sections, which were stained with JIM5 antibodies. Images were obtained by merged signals from JIM5, DAPI staining (blue color), and chlorophyll autofluorescence. Green fluorescence indicates the presence of low-methylated HG. C, cuticle; E, epidermis; M, mesophyll; Ph, phloem; Sch, substomatal chamber; Vb, vascular bundles; X, xylem. Bars = 50 μm **(A,B)**, 15 μm **(C,D)**.

**FIGURE 8:**
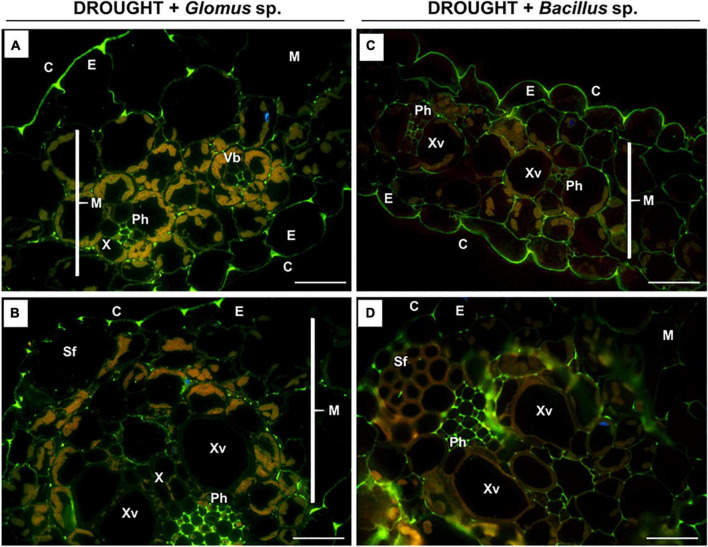
Immunodetection of low-methylated homogalacturonans (HG) in the leaves of drought-stressed *Z. mays* pretreated with *Glomus* sp. or *Bacillus* sp. strains. Reactions with monoclonal antibody JIM5 were made on leaf sections cut from drought-stressed maize treated at the seed stage with *Glomus* sp. **(A,B)** or *Bacillus* sp. **(C,D)**. Images were obtained by merged signals from JIM7, DAPI staining (blue color), and chlorophyll autofluorescence. Green fluorescence corresponds to the presence of low-methylated HG. C, cuticle; E, epidermis; M, mesophyll; Ph, phloem; Sf, sclerenchyma fibers; Vb, vascular bundles; X, xylem. Bars = 15 μm.

Based on the results of ruthenium red staining, in the next step, we analyzed more comprehensively the degree of pectin methylation in the leaves of treated plants by immunolabeling with the monoclonal antibodies JIM7 (Figures 5, 6) and JIM5 (Figures 7, 8). Fluorescence indicating the presence of high-methylated pectins under control conditions was observed in the upper and lower layers of mesophyll cells located directly under the epidermis (Figure 5A). Higher magnifications revealed that the signal was emitted by cell walls (Figure 5B). Water deficit changed the localization pattern of these pectins and resulted in their distribution throughout the mesophyll area (Figure 5C). Furthermore, they accumulated more strongly when compared to the sections obtained from well-watered plants (Figure 5D). In both the control and the drought-treated leaves, no fluorescence was observed in the area of the vascular bundles (Figures 5A,C).

When *Z. mays* seeds were inoculated with *Glomus* sp. or *Bacillus* sp. and subjected to drought, highly esterified HGs were redistributed in the leaf tissues (Figure 6). In both cases, a relatively high signal after reaction with the JIM7 antibody was emitted by cell walls of the epidermis, mesophyll, and vascular tissues (Figures 6A,C). More specifically, the application of *Glomus* sp. resulted in the accumulation of JIM7-dependent fluorescence in the epidermis tissue, elements of phloem and xylem, and sclerenchyma fibers (Figure 6B). Simultaneous treatment of maize with drought and *Bacillus* sp. caused preferential localization of highly methylated pectin in mesophyll and epidermis (Figure 6C). Furthermore, strong fluorescence was detected in the cell walls of vessels, xylem and phloem, and epidermis cells adjacent to the vascular bundles (Figure 6D).

The fluorescence signals detected in the leaves after incubation with JIM5 were different under control and drought conditions (Figure 7). In general, the distribution of low-methylated pectin in the stressed leaf was homogenous throughout the mesophyll tissue adjacent to the epidermis (Figure 7C). Strong labeling was noted in the intracellular spaces of cells specifically disrupted by drought (Figure 7D). Such strong staining was not visible in the control section; however, weak fluorescence was localized in the mesophyll cell wall (Figure 7A), particularly around the vascular region (Figure 7B).

As evident in Figure 7, a similar localization pattern of low-methylated pectins characterized drought-stressed leaves of *Z. mays* subjected to the action of *Glomus* sp. or *Bacillus* sp. The highest signal density was observed in the epidermis cells (Figures 8A,C) and phloem located in the central leaf vasculature (Figures 8B,D). Low labeling, manifested by several luminous spots or short bands in the cell walls, characterized the mesophyll tissue (Figures 8A–D).

## Discussion

Maize is extremely sensitive to drought, given the high demand for water, particularly in the stage of vegetative development and during grain filling (Rafique, 2020). This stress leads to morphological and anatomical changes, influences cell structure, and induces multiple metabolic pathways. Water deficit reduces maize growth (Abrecht and Carberry, 1993), leaf area (NeSmith and Ritchie, 1992; Çakir, 2004), water content, and photosynthesis-related parameters (NeSmith and Ritchie, 1992). All these modifications extremely influence the yield of the crop. To improve its cultivation, we need to understand how maize recognizes drought conditions at the level of each organ and develops agrotechnical approaches aimed to improve the resistance of this species to water deficit. Nowadays, more emphasis should be given to the elimination of pesticides and the search for new biologically active, high-value substances produced by microorganisms. Among them, strains of *Glomus* sp. should be considered. Their activity in the reduction of stress effects has been proven in lettuce (Ruiz-Lozano et al., 1995b), *Lavendula spica* (Marulanda et al., 2007), and *Cinnamonum migao* (Liao et al., 2021). Another natural solution for the improvement of plant tolerance to unfavorable conditions is inoculation with different strains of *Bacillus* sp., as revealed in *Capsicum annuum* (Lim and Kim, 2013), *Z. mays* (Moreno-Galván et al., 2020), *Solanum lycopersicum* (Gowtham et al., 2020), *Glycine max* (Sheteiwy et al., 2021), *Cenchrus americanus* (Kushwaha et al., 2020), and *Helianthus annus* (Sandhya et al., 2011). Additionally, Marulanda et al. (2006) demonstrated that interactions between *Bacillus thuringiensis* and *Glomus* increase plant water uptake in *Retama sphaerocarpa* under drought. Given the high potential relevance of these microorganisms, in this paper, we checked whether inoculation of maize seeds with *Glomus* sp. or *Bacillus* sp. alleviates drought-evoked effects in leaves and, in this way, limits its negative effects on vegetative development. The changes observed in Fv/Fm in this study suggest that both microorganisms improved the efficiency of photosynthetic apparatus under drought stress (Figure 1A). Similar to the results of our experiment, *Glomus* increased PSII-effective efficiency in salt-stressed maize (Xu et al., 2018). Moreover, literature data provide evidence that *Bacillus* improved Fv/Fm in *Capsicum chinensis* (Samaniego-Gámez et al., 2021) and *Euterpe oleracea* (Castro et al., 2020). Furthermore, the Fv/Fm ratio of *Solanum lycopersicum* was reduced when three *Bacillus* spp. were applied (Costa-Santos et al., 2021). Furthermore, *Bacillus amyloliquefaciens* mixed with *Azospirillum brasilense* NO40 increased the photosynthetic rate of *Triticum aestivum* (Kasim et al., 2013).

Inoculation of *Z. mays* with *Glomus* sp. or *Bacillus* sp. reduces the content of proline under drought; however, its level was higher than in control (Figure 2). Liao et al. (2021) suggested that *Glomus* improves the growth of *Cinnamonum migao* through better absorption of nutrients and water uptake, ensuring a high turgor of tissues, and thus it is not necessary to synthesize large amounts of osmoprotectants. However, different *Bacillus* strains increased proline secretion in drought conditions in *Solanum lycopersicum* (Shintu and Jayaram, 2015), *Cicer arietinum* (Sharma et al., 2013), *Sorghum bicolor* (Grover et al., 2014), and *Cucumis sativus* (Wang et al., 2012). We propose, based on our results, that the reduced content of proline in stressed and inoculated maize when compared to drought-treated plants might be related to their increased tolerance to drought evoked by the action of *Bacillus* sp. and *Glomus* sp.

The reduced efficiency of the photosynthetic apparatus could be a result of the production of ROS due to changes in the electron transport, which is reflected by the decreased pool size of electron acceptors (Reddy et al., 2004). Moreover, ROS can initiate lipid peroxidation and induce cell membrane destruction (Sabra et al., 2012).

In *Z. mays*, water deficit in soil promoted the formation of ROS, including O_2_^•⁣–^ and H_2_O_2_ (Figures 2A,C), and caused membrane destabilization, which is reflected by the accumulation of MDA, as a marker (Figure 1C). Inoculation of seeds with *Glomus* sp. or *Bacillus* sp. mitigated this harmful effect of drought (Figure 3), since we observed that reduced content of O_2_^•⁣–^ (Figure 2A) correlated with decreased SOD activity (Figure 2B). Another symptom of oxidative stress neutralization by *Glomus* sp. and *Bacillus* sp. in maize is also evident by a reduction in H_2_O_2_ (Figure 2C) level and, consequently, the activity of CAT (Figure 2D). Literature data suggest that *Glomus* can act on the antioxidant enzymes of plants in a species-dependent manner. Generally, these fungi accelerate the activity of antioxidant enzymes in plants under drought, for instance, POX in *Juglans* (Behrooz et al., 2019) and *Citrus tangerine* (Wu et al., 2007), CAT in *Cinnamomum migao* (Liao et al., 2021), SOD and CAT in foxtail millet (Gong et al., 2015), SOD and POX in bean (Ganjeali et al., 2018), and CAT, APX, and POX in *Triticum aestivum* (Yaghoubian et al., 2014). In foxtail millet, *Glomus intraradices* decreased the concentration of H_2_O_2_ and O_2_^•⁣–^, compared with non-inoculated plants (Gong et al., 2015). Furthermore, *G. mosseae* reduced the level of H_2_O_2_ in wheat (Yaghoubian et al., 2014). Several studies, similar to the one presented herein, provide evidence that treatment of plants with *Bacillus* negatively influenced the activity of the antioxidant system, e.g., in *Triticum aestivum* (Kasim et al., 2013) and tomato (Arias Padró et al., 2021). Nevertheless, another relationship was observed in potatoes, in which CAT, APX, and SOD were upregulated when plants were inoculated with *Bacillus pumilus* and *Bacillus firmus* (Gururani et al., 2013). The negative impact of *Glomus* sp. and *Bacillus* sp. on the ROS content and antioxidant enzyme activities in drought-treated maize observed in this study strongly suggest that these microorganisms alleviate adverse effects of water deficit related to ROS burst. Such a hypothesis is supported by a decreased level of MDA (Figure 1C) in the leaf of maize inoculated with *Glomus* sp. or *Bacillus* sp., given that a high amount of MDA is derived from the lipid peroxidation of polyunsaturated fatty acids which is induced by ROS. Similar observations were noticed for MDA content in drought-stressed foxtail millet inoculated with *Glomus* (Gong et al., 2015), as well as in *Solanum lycopersicum* and cucumber treated with various *Bacillus* strains under drought conditions (Wang et al., 2012; Gowtham et al., 2020).

Lipid peroxidation indicates cell membrane rupture, which is visible in cellular structure. Indeed, microscopy analysis showed shrinking protoplasts of mesophyll cells in maize leaves (Figure 3) as a result of decreased water potential in cells under drought due to its limited availability. This also results in the accumulation of osmoprotective substances, such as proline (Figure 1B). What is more, a reduction in the diameter of the xylem vessels (Figure 3F) might be related to the disrupted water transport due to reduced hydraulic conductivity. The decreasing turgor pressure of mesophyll and xylem cells under drought affects their expansion, disrupting their architecture, so these tissues adapt their anatomy to environmental conditions and ensure long-distance transport (Abe et al., 2003). Reduced xylem size, as an effect of drought, was observed in *Pyrus communis* (Barss, 1930), *H. annuus, T. aestivum* (Penfound, 1931), and *Ricinus communis* L. (Penfound, 1932). The observed reduction in photosynthetic activity (Figure 1A) in this study might be related to drought-triggered modifications in chloroplast localization and structure (Figures 3F,J). The above-described symptoms were not associated with stress responses in plants inoculated with *Glomus* sp. or *Bacillus* sp., with better effects observed when bacteria were used (Figures 3G,H).

Modifications in the cell structure caused by drought (Figure 3) suggest changes in the cell wall structure. In the cell wall, hemicelluloses can bind to lignin and cellulose to improve cell wall rigidity, which strengthens this structure (Le Gall et al., 2015). So, the decrease in hemicellulose level in maize under drought might indicate a loss of cell wall integrity (Figure 4A). Such observations were also demonstrated in *Arabidopsis*, tobacco suspension cells, grape leaves, and wheat roots under drought (Feng et al., 2016). However, accumulated hemicelluloses may break, especially under stress conditions, thus preserving the plasticity of the wall structure (Le Gall et al., 2015; Tenhaken, 2015). Therefore, the increasing level of hemicelluloses under drought in maize inoculated with microorganisms (Figure 4A) may be a manifestation of a structural adjustment to combat the effects of stress.

Another manifestation of cell wall remodeling under drought is increasing pectin levels (Figure 4B). One of the plant’s protective mechanisms under drought conditions is the synthesis of pectins, which can form protective colloids due to their ability to bind water (Wu et al., 2018). However, the content of these compounds in the inoculated maize, despite the drought action, was the same as in the unstressed control plants (Figure 4B). A possible reason is the alleviation of drought stress by microorganisms, so the plant does not accumulate pectin.

The biomechanical properties of the cell wall, which are crucial for the modulation of its structure under drought, are determined by the methylation of pectins, e.g., homogalacturonans (HGs) (Forand et al., 2022). The synthesis of a highly esterified HG takes place in the Golgi apparatus, and then they are exported into the cell wall and de-esterified by pectin methylesterase (Willats et al., 2001). High- and low-methylated HG are accumulated in the leaves of drought-stressed maize (Figures 5, 7), indicating both the synthesis and de-esterification of these compounds. A stronger effect observed in the case of low-methylated HG under stress (Figures 7C,D) is the argument for the reduced plasticity and loosening of the wall structure. Furthermore, inoculation of maize with *Glomus* sp. or *Bacillus* sp. had not changed the general pool of pectin (Figure 4B), however, affected the degree of HG methylation and their distribution in leaf cells (Figures 6, 8). These results indicate that both microorganisms caused an intensive reorganization of the cell wall structure.

Low-methylated HG in the leaves of inoculated maize presented in the mesophyll cells, epiderm, and vascular bundles, particularly phloem (Figure 8). Pectin de-esterification could be a defense reaction of the plant leading to the generation of free carboxyl groups and the formation of gels by binding Ca^2+^ ions, which consequently leads to the appearance of a cross-link that stabilizes and mechanically strengthens cell walls (Willats et al., 2001; Braybrook et al., 2012). In the case of phloem, such a strategy enables the plant to efficiently uptake water and mineral compounds, as well as those involved in osmoregulation. High content of low-methylated HG in the phloem of stressed plants and plants inoculated with microorganisms (Figure 8) might protect against the deformation of cells characteristic of drought-treated leaves (Figures 3B,F,J). On the other hand, the accumulation of high-methylated HG in the leaves of stressed and inoculated maize (Figure 6) supports the *de novo* formation of these compounds. There is a report showing that salt-tolerant genotypes of *Z. mays* are characterized by an increased content of these pectins (Uddin et al., 2013). The appearance of methylated HG in the epidermis ensures strength and elasticity of the cell wall, which is important in the adaptation to changing turgor pressure by stomatal movements under stress conditions (Jones et al., 2005). Therefore, maintaining the cohesion and appropriate flexibility of the cell wall mediated by the balance between high- and low-methylated pectins (Figure 6, 8) could be a part of the mechanism induced by *Glomus* sp. and *Bacillus* sp. to protect maize against drought stress consequences.

**FIGURE 9:**
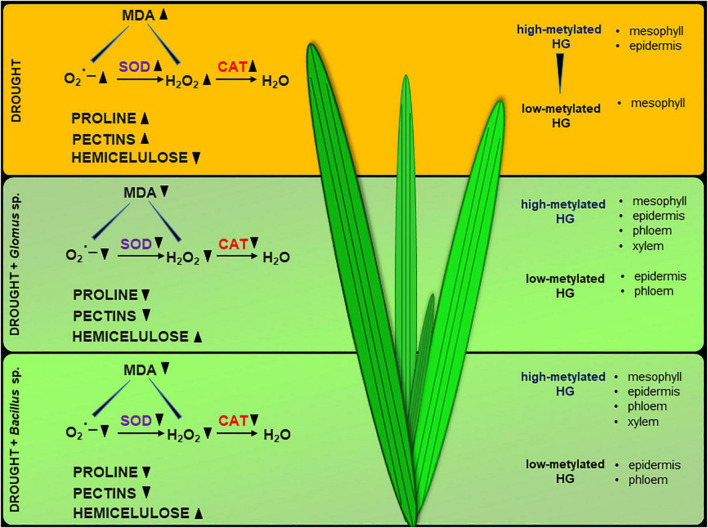
Possible action of *Glomus* sp. and *Bacillus* sp. in the improvement of soil drought tolerance in maize. The scheme was prepared based on the obtained here results.

Collectively, our results support that *Glomus* sp. and *Bacillus* sp. help *Z. mays* to cope with drought stress, since inoculation of the seeds with these microorganisms prevents inhibition of photosynthesis and disruption in redox balance. Based on the presented observations, we suggest that *Glomus* sp. and *Bacillus* sp. modify the cell wall structure of maize leaves by affecting the pectin methylation level and hemicellulose content (Figure 9). It could lead to alleviation of the negative effects of drought in this species. We provide novel insight into drought stress resistance in important crop species, which could be helpful for agriculture and biotechnology development.

## Data availability statement

The original contributions presented in this study are included in the article/supplementary material, further inquiries can be directed to the corresponding author.

## Author contributions

EW and AK conceived and designed the research, conducted the experiments, evaluated and analyzed the data, and wrote and completed the manuscript. MWo was responsible for plant cultivation, material collection, photosynthesis analyses, and helped with spectrophotometric analyses. MŚ prepared sections for microscopy. KB was involved in the immunolocalization experiments. KB, AB-B, and MWa organized the tools and media for microorganisms and were responsible for bacterial and fungal growth, and reviewed the manuscript. All authors contributed to the article and approved the submitted version.
